# Ketogenic diet: new avenues to overcome colorectal cancer

**DOI:** 10.1038/s41392-022-01113-9

**Published:** 2022-08-02

**Authors:** Yuancai Xiang, Meng Wang, Hongming Miao

**Affiliations:** 1 Department of Biochemistry and Molecular Biology, Third Military Medical University (Army Medical University), Chongqing, 400038 China; 2 Department of Biochemistry and Molecular Biology, Southwest Medical University, Luzhou, 646000 China

**Keywords:** Cancer therapy, Gastrointestinal cancer

**Fig. 1:**
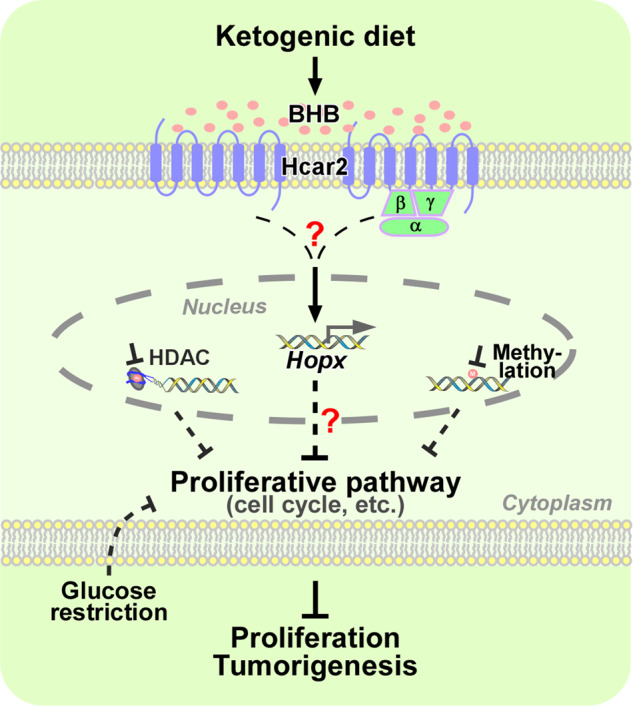
Schematic illustration of the molecular mechanisms underlying KD-mediated suppression in CRC growth. During a ketogenic diet, abundant ketone bodies, especially BHB, were produced in the liver and were transferred to colorectum via blood circulation. Once the concentration of BHB elevated around colorectal tumor cells, it binds to Hcar2, a G-protein-coupled receptors, and activation Hopx expression, thereby inhibiting cell proliferation and tumorigenesis through suppressing proliferative pathway, such as cell cycle. In addition, multiple combination treatment based on KD demonstrated additive effects on the suppression of CRC, such as the combination with glucose restriction, histone deacetylases inhibitors, and DNA methylation inhibitor. BHB: β-hydroxybutyrate; Hcar2: hydroxyl-carboxylic receptor 2; α, β, γ: G-protein subunits; Hopx: homeobox only protein homeobox; HDAC: histone deacetylases

In a recent article published in *Nature*, Dmitrieva-Posocco et al.^1^ showed that ketogenic diets (KD) exert strong abilities to prevent and treat colorectal cancer (CRC) via the ketone body β-hydroxybutyrate (BHB)-mediated hydroxyl-carboxylic receptor.^2^ (Hcar2)-homeobox only protein homeobox (Hopx) signaling axis in multiple in vivo and in vitro models (Fig. 1). Intervention of this signaling axis through different settings can restrict the growth of CRC, which provides us a new insight for ketogenic diet-based CRC treatment.

CRC is one of the most common malignant tumors in the world that threaten human health. Multiple meta-analyses have shown that a posteriori–derived unhealthy dietary pattern associated with higher body mass index and energy intake increased the risk of colon cancer,^2^ such as starchy foods, sugary drinks, salty snacks, red and processed meat, and refined carbohydrates. As Hippocrates said, ‘Let food be thy medicine and medicine be thy food’. Changing dietary pattern at different periods of treatment could indeed improve the therapeutic effect, such as fasting^3^ and high-fat diet.^4^ Even so, diet-based strategies for CRC prevention and therapy remain largely unclear. In this study, Dmitrieva-Posocco et al. detected the effect of dietary intervention on CRC growth by feeding designed mouse diets containing constant protein and different fat-to-carbohydrate ratios in azoxymethane/dextran sodium sulfate (AOM/DSS)-induced model and genetic mice model (Cdx2^creERT^APC^fl/fl^) in different housing conditions. They observed an inhibitory effect that enhanced gradually with the increase of fat-to-carbohydrate ratios, especially in the KD group including 90% fat content regardless of its origin. Moreover, substitution normal diet with KD during the process of AOM/DSS-based model construction also suppressed CRC growth, whereas this effect disappeared while return to a normal diet.

To uncover the underlying mechanisms of how KD inhibits CRC growth, the authors investigated the role of two identified KD-inhibited factors, IL-17-producing immune cells and NLRP3 inflammasome, in AOM/DSS models with *Rag1*^*−/*−^ mice and *NLRP3*-deficient bone marrow chimeric mice, respectively. However, the tumor suppression of KD did not obviously change. In fact, immunohistochemical stain demonstrated an overall decreased turnover of intestinal epithelial cells (i.e., increased proliferation and decreased apoptosis) in both healthy and CRC burden mice. Thus, they applied organoid system and co-culture method and found that KD decelerated cell renew via suppressing the function of Lgr5^+^ stem cells that resided in intestinal crypt. These results revealed the target cells of KD in CRC treatment. Furthermore, the authors evaluated the role of typical metabolites of KD, acetoacetate and BHB, in epithelial cells growth through organoid culture, and demonstrated that only BHB could inhibit intestinal organoid growth in a concentration-dependent manner. Of note, the effective concentration of BHB in vitro setting is similar to the levels in the gut of KD feeding mice, and tumor-derived organoids were more sensitive to BHB than that in wild-type organoids. Interestingly, the concentration of BHB in vivo did not affect cell activity in normal organoids, indicating that BHB is an effective metabolite of KD in CRC treatment. This hypothesis was further confirmed by elevating serum BHB concentration with oral BHB esters or delivery BHB salts using osmotic mini-pumps, implying that utilization of BHB to restrict the CRC progression has strong operability in clinical practice.

Besides as a carrier of energy, BHB, the most abundant ketone body in mammals, exerts multiple functions in maintaining cellular steady state, including lipid metabolism, metabolic rate, and gene expression.^5^ To reveal the downstream target of BHB, the authors employed RNA sequencing (RNA-seq) to screen a tumor negative regulator homeobox only protein homeobox (Hopx) in BHB-treated organoids. Intervention of Hopx expression through knock-out or overexpression indeed modulated the inhibitory effect of BHB on organoid growth. Moreover, the suppressive effect of KD was lost in CRC-beared Hopx-deficient mice, accompanied by activation of several proliferative-related pathways. These data indicated that Hopx is a vital downstream target of BHB for performing tumor inhibition. Furthermore, they try to reveal the detailed mechanism of how Hopx was elevated by BHB, however, two typical factors of transcriptional regulation, histone deacetylation and DNA methylation, were ruled out via comparing the results of RNA-seq or ChIP-sequencing and bisulfite sequencing, respectively, between inhibitor-treatment groups and BHB-treatment group, although the inhibitors of histone deacetylation and DNA methylation inhibited organoid growth. Then, seven candidate downstream genes of BHB were screened by organoid-based CRISPR system. Among these genes, only Hcar2, a BHB receptor, deficient organoid demonstrated strong resistance to BHB-treatment with a failed activation of Hopx. These results suggest that Hcar2 acts as an indispensible mediator in BHB-Hopx axis for exerting tumor-suppressive effect. More importantly, the role of BHB-Hopx pathway in CRC treatment was also found in organoids cultured with human healthy or patient intestinal crypts, and clinical data displayed a strong relevance between serum BHB, colonic *Hopx* levels, and cell cycle, which implied that manipulation of BHB-Hopx pathway may be a potential effective way for CRC treatment.

In summary, the work by Dmitrieva-Posocco et al. revealed a novel mechanism that KD constrains CRC growth via activation of the BHB-Har2-Hopx axis, which provides new insight for CRC therapy. However, detailed mechanisms of how Har2 regulates *Hopx* expression to further modulate the proliferative signaling pathway remains unclear (Fig. 1). In addition, on the way to the identification of this signaling axis, multiple combination treatment based on KD in this work also demonstrated additive effects in tumor suppression with different mechanisms, such as the combination with glucose restriction, histone deacetylase inhibitors (vorinostat and butyrate) and DNA methylation inhibitor (5-azacitidine). Moreover, given that Hopx was specifically elevated in colonic tissue of KD-fed mice, and could respond to BHB only in a few CRC cell lines, together with the actuality that KD has been clinically applied in epilepsy for many years, KD-based multiple combination strategies offer a variety of options for personalized treatment in patients with CRC, especially in the type of BHB-Hcar2-Hopx axis high responsiveness.
